# Robot-Assisted Minimally Invasive Esophagectomy with Intrathoracic Anastomosis (Ivor Lewis): Promising Results in 100 Consecutive Patients (the European Experience)

**DOI:** 10.1007/s11605-019-04510-8

**Published:** 2020-02-18

**Authors:** Pieter Christiaan van der Sluis, Evangelos Tagkalos, Edin Hadzijusufovic, Benjamin Babic, Eren Uzun, Richard van Hillegersberg, Hauke Lang, Peter Philipp Grimminger

**Affiliations:** 1 Department of General, Visceral and Transplant Surgery, University Medical Center of the Johannes Gutenberg University, Langenbeckstrasse 1, D-55131 Mainz, Germany; 2 Department of Surgery, University Medical Center Utrecht, Utrecht, Netherlands

**Keywords:** Esophageal cancer, Minimally invasive, MIE, RAMIE, Ivor Lewis

## Abstract

**Background:**

Robot-assisted minimally invasive esophagectomy (RAMIE) with intrathoracic anastomosis is gaining popularity as a treatment for esophageal cancer. The aim of this study was to describe postoperative complications and short-term oncologic outcomes for RAMIE procedures using the da Vinci Xi robotic system 4-arm technique.

**Methods:**

Data of 100 consecutive patients with esophageal or gastro-esophageal junction carcinoma undergoing modified Ivor Lewis esophagectomy were prospectively collected. All operations were performed by the same surgeon using an identical intrathoracic anastomotic reconstruction technique with the same perioperative management. Intraoperative and postoperative complications were graded according to Esophagectomy Complications Consensus Group (ECCG) definitions.

**Results:**

Mean duration was 416 min (±80); 70% of patients had an uncomplicated postoperative recovery. Pulmonary complications were observed in 17% of patients. Anastomotic leakage was observed in 8% of patients. Median ICU stay was 1 day and median overall postoperative hospital stay was 11 days. The 30-day mortality was 1%; 90-day mortality was 3%. A R0 resection was reached in 92% of patients with a median number of 29 dissected lymph nodes. All patients had at least 7 months of follow-up with a median follow-up of 17 months. Median overall survival was not reached yet.

**Conclusion:**

RAMIE with intrathoracic anastomosis (Ivor Lewis) for esophageal or gastro-esophageal junction cancer was technically feasible and safe. Postoperative complications and short-term oncologic results were comparable to the highest international standards nowadays.

## Synopsis

RAMIE with intrathoracic anastomosis (Ivor Lewis) for esophageal or gastro-esophageal junction cancer is technically feasible and safe. Postoperative complications and short-term oncologic results were comparable to the highest international standards nowadays.

## Introduction

Esophageal cancer is the sixth leading cause of death from cancer worldwide with an estimated 400.000 new cases annually.^1^ Multimodality therapy combined with a transthoracic esophagectomy with 2-field lymph node dissection is currently the standard treatment with curative intent for patients with esophageal cancer or cancer of the gastro-esophageal junction (GEJ).^2–4^

Minimally invasive esophagectomy was designed to reduce the surgical trauma of the open transthoracic esophagectomy (OTE).^5^ MIE improved postoperative outcomes compared to open transthoracic esophagectomy (OTE).^6–8^ Until now, there is randomized controlled trial (TIME trial) which compared conventional minimally invasive esophagectomy to open transthoracic esophagectomy (OTE).^6^ MIE resulted in a lower incidence of respiratory infections compared to OTE with better quality of life, even after 1 year.^6,7^

Robot-assisted minimally invasive esophagectomy (RAMIE) was developed to overcome the technical limitations of conventional MIE, such as 2-dimensional vision and rigid instruments.^9,10^ RAMIE facilitates complex minimally invasive procedures with an enlarged 3-dimensional view, and the articulated instruments allow dissection with 7 degrees of freedom, which is beneficial for the precise dissection in the posterior mediastinum. RAMIE was shown to be technically feasible and safe in terms of postoperative complications and oncological outcomes.^9,10^

There is 1 randomized controlled trial (ROBOT trial), which compared RAMIE to open esophagectomy (OTE).^8^ In this randomized controlled trial, the da Vinci Si robotic system was used, and all patients received a cervical end-to-side hand-sewn anastomosis (McKeown). RAMIE resulted in a lower percentage of overall surgery-related and cardiopulmonary complications with lower postoperative pain and better short-term quality of life with better short-term postoperative functional recovery compared to OTE.^8^ Oncologic outcomes, such as the percentage of radical resections (R0), the number of resected lymph nodes, and disease-free and overall survival, were comparable between RAMIE and OTE. All oncologic outcomes were comparable to the highest standards worldwide nowadays.^8^

The RAMIE technique was introduced within our hospital under supervision of experienced RAMIE surgeons, using a structured proctoring program to pass the learning curve faster without compromising surgical quality.^11^ However, the original RAMIE technique underwent some technical modifications; the 4-arm da Vinci Xi robotic system was used, and currently a circularly stapled intrathoracic anastomosis is performed.^12,13^ An intrathoracic anastomosis (Ivor Lewis) might be associated with a lower incidence of anastomotic leakage, 90-day mortality, and postoperative morbidity compared to a McKeown procedure.^14^ The RAMIE technique was shown to be technically feasible and safe.^13^

In this article, we present our first 100 consecutive and unselected cases of RAMIE with an intrathoracic anastomosis using the da Vinci Xi robotic system (Ivor Lewis) using a circular stapler with 4 robotic arms. The aim of this study was to describe postoperative complications and short-term oncologic outcomes for RAMIE4.

## Methods

### Patients

Between January 2017 and February 2019, 143 consecutive patients with esophageal cancer or cancer at the gastro-esophageal junction were eligible for a transthoracic esophagectomy with intrathoracic anastomosis in the University Medical Center of the Johannes Gutenberg University (Mainz, Germany). Out of 143 patients, 39 patients underwent conventional MIE (27.3%), 3 patients underwent hybrid esophagectomy (2.1%), and 1 patient underwent open transthoracic esophagectomy (0.7%), leaving 100 patients eligible for RAMIE (69.9%). The selection for RAMIE patients was dependent on the availability the Xi da Vinci robotic system (da Vinci Xi system, Intuitive Surgical Inc. Sunnyvale, CA, USA) 1 day a week, and there was no patient selection.

Data on surgical procedures and postoperative outcomes were registered prospectively in an institutional database and were discussed in a weekly meeting. Patient- and treatment-related characteristics were prospectively collected.

Outcome data included operative times of the abdominal and thoracic phase of the operation, blood loss, length of ICU stay, total hospital stay, and 30-day and 90-day mortality. Intraoperative and postoperative complications were graded according to definitions stated by the Esophagectomy Complications Consensus Group (ECCG).^15^

Patients received postoperative follow-up at the outpatient department according to the standard follow-up regimen described in the German guidelines. This prospective study was approved by the institutional review board of the Johannes Gutenberg University (Mainz, Germany), and the requirement to obtain informed consent was waived. Initial staging included endoscopy combined with endoscopic ultrasonography and tumor biopsy followed by a computed tomography scan of the abdominal and thoracic region.

Prior to treatment, all patients were discussed in an upper gastrointestinal multidisciplinary tumor board to determine optimal treatment. Our cancer center followed the guidelines of the German Cancer Society (DKG) and is board certified (quality control was performed every year). Positron emission tomography (PET) scans were not routinely used in the work up of patients, only when distant metastases were suspected.

The standard neoadjuvant treatment for patients with esophageal adenocarcinoma was perioperative chemotherapy with FLOT (4 preoperative and 4 postoperative 2-week cycles of docetaxel 50 mg/m^2^, intravenous oxaliplatin 85 mg/m^2^, intravenous leucovorin 200 mg/m^2^, and fluorouracil 2600 mg/m^2^ as a 24 h infusion) or CROSS [preoperative chemoradiotherapy with carboplatin (area under the curve of 2 mg per milliliter/min) and paclitaxel (50 mg/m^2^ of body surface area) for 5 weeks and concurrent radiotherapy (41.4 gray in 23 fractions, 5 days per week)].^16,17^ All patients with esophageal squamous cell carcinoma received CROSS. All cT1N0 patients who underwent esophagectomy had an incomplete endoscopic mucosal resection (R1) or endoscopic submucosal dissection before and did not undergo any neoadjuvant treatment.

### Perioperative Management

A gastroscopy with pylorus dilatation was conducted 1 day before the operation to prevent postoperative delayed gastric emptying.^18^ All patients received an epidural catheter and were intubated with a left-sided double-lumen tube. Antibiotic prophylaxis (ampicillin 2000 mg and sulbactam 1000 mg) was administered 30 min prior to incision. Postoperatively, all patients were extubated in the operating theater and were admitted to the intensive care unit (ICU) hereafter. Hemodynamical and respiratory stable patients were discharged towards the surgical ward. NO feeding tubes were placed. All patients were placed on a nil-by-mouth routine for the first 3 days postoperatively. In absence of clinical signs of anastomotic insufficiency, patients started with sips of water, and the oral intake was gradually increased to solid food. Postoperative esophageal swallow tests were not routinely performed. There was no enhanced recovery program.

### RAMIE4 Operating Technique

The RAMIE4 operating technique was described before.^12,13^ For the abdominal phase of the procedure, the patient was placed in supine position. The stomach was mobilized, and a gastric conduit was created robotically. An abdominal lymph node dissection was performed, including lymph nodes located at the common hepatic artery, portal vein, left gastric artery, splenic artery, and the suprapancreatic and lesser omental lymph nodes.^12,13^

For the thoracic part of the procedure, the patient was positioned in semi-prone position. The thoracic esophagus was mobilized, and a thoracic lymphadenectomy was performed.^12,13^ Hereafter, the esophagus was transected above the azygos vein. The purse string suture in the esophageal stump was performed robotically using a Prolene 2/0 90 cm (Ethicon, USA). The assistant trocar was widened to create a mini-thoracotomy. The 25 or 28 mm stapler head of the circular stapler (DST Series EEA, Medtronic, USA) was inserted though the mini-thoracotomy into the esophageal stump, and the purse sting suture was knotted manually. The esophageal specimen was pulled up, and a circular anastomosis was created. The surplus of the gastric conduit was stapled, and the resection specimen was removed through the mini-thoracotomy. A V-Loc (Medtronic, USA) running suture was used to oversew the circular anastomosis. An omental wrap was placed around the anastomosis (270° or 360°). One right-sided chest tube was inserted.^12,13^

### Pathological Analysis

The resected specimen was evaluated using a standard protocol. The 8th edition of the International Union Against Cancer (UICC) was used for Tumor Node Metastasis (TNM) classification, tumor grade, and stage grouping.^19^ The (circumferential) resection margins were evaluated using the College of American Pathologist (CAP) criteria.^20^

### Statistical Analysis

Statistical analysis was performed using SPSS version 25.0 (SPSS, Chicago, IL, USA). We considered a *P* value of < 0.05 to be statistically significant. All normally distributed continuous data were presented as means with standard deviations. All skewed continuous data were presented as medians with range. Overall survival (OS) was calculated from the date of surgery to the date of death or last follow-up.

## Results

Baseline data, demographics, and tumor characteristics were demonstrated in Table 1. Our collective consisted of 91 male and 9 female patients, with a median age of 61 years (range 26–86). In total, 20 patients were staged with a cT1-cT2 disease. The remaining 80 consisted of 72 cT3 patients and 8 patients with cT4 tumors. In the preoperative staging, 45 patients were found to have nodal positive disease. Most of the tumors (59%) were localized in the middle esophageal. A third of the patients did not suffer from comorbidities (34%). Prior thoracic and abdominal operations were performed in 26 patients. None of the patients received an esophageal or gastric operation. Most of the patients received neoadjuvant chemoradiotherapy (44%) or perioperative chemotherapy (39%).

**Table 1:** Patient demographics and tumor characteristics (*n* = 100)

	n = 100
Age (y) (median – range)	61 (26–86)
Gender (n (%))	
M	91 (91)
F	9 (9)
BMI (kg / m^2^) (median – range)	25.4 (14.9–46.3)
Comorbidity (n (%))	
No comorbidity	34 (34)
Vascular	39 (39)
Cardiac	23 (23)
Diabetes	13 (13)
Pulmonal	20 (20)
Oncologic	7 (7)
Previous thoracic/abdominal operation	26 (26)
ASA score (n (%))	
2	49 (49)
3	47 (47)
4	4 (4)
Clinical stage (TNM 8) (n (%))	
cT1aN0	4 (4)
cT1aN1	1 (1)
cT1bN0	3 (3)
cT2N0	7 (7)
cT2N1	2 (2)
cT2N2	3 (3)
cT3N0	36 (36)
cT3N1	24 (24)
cT3N2	10 (10)
cT3N3	2 (2)
cT4aN0	5 (5)
cT4aN1	2 (2)
cT4aN2	1 (1)
Tumor location (n (%))	
Upper esophageal	2 (2)
Middle esophageal	10 (10)
Lower esophageal	59 (59)
GEJ	29 (29)
Tumor type (n (%))	
Adenocarcinoma	79 (79)
Squamous cell carcinoma	19 (19)
Melanoma	1 (1)
Neuroendocrine	1 (1)
Neoadjuvant treatment (n (%))	
No therapy	16 (16)
Chemotherapy	39 (39)
Chemoradiotherapy	44 (44)
Radiotherapy	1 (1)

**Fig. 1:**
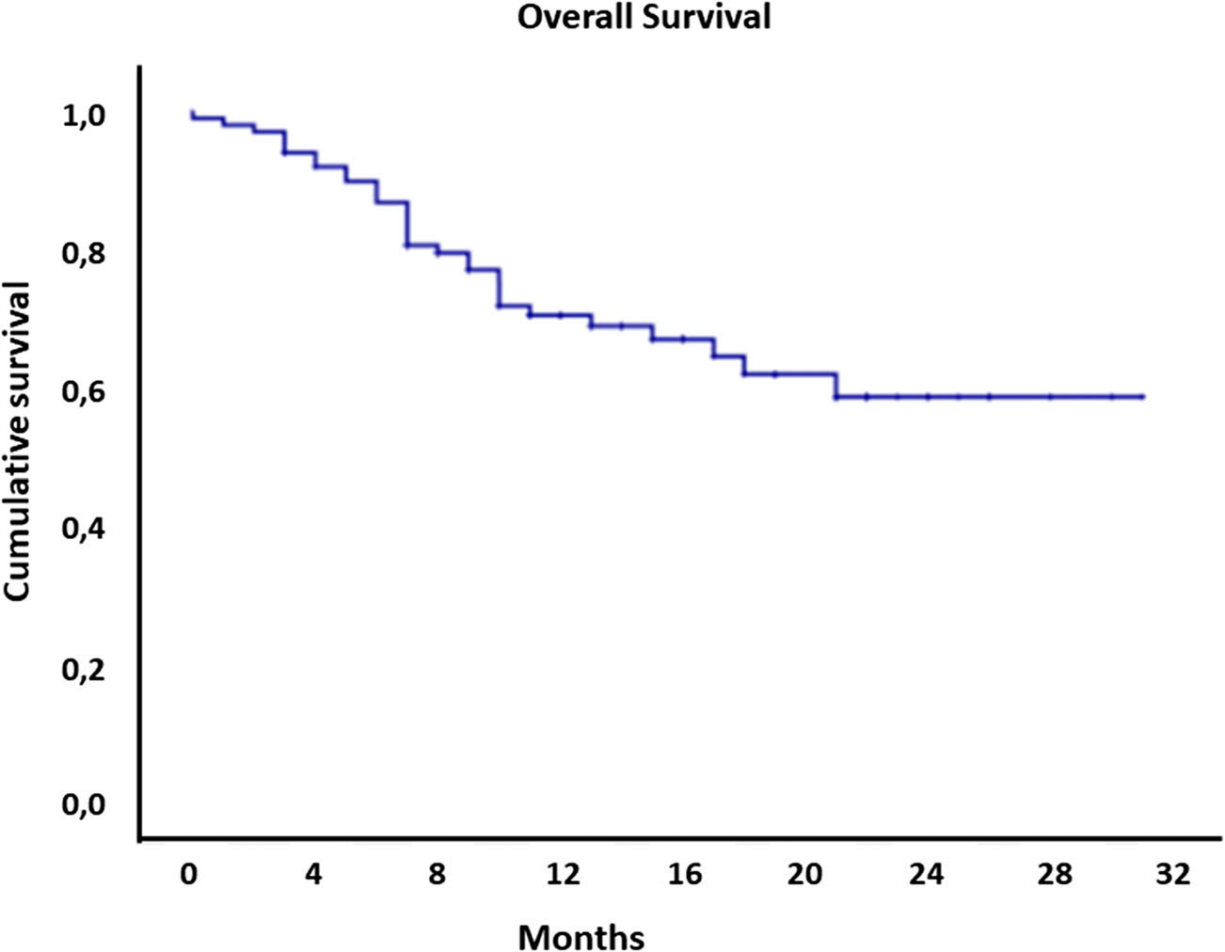
Overall survival for RAMIE (Ivor Lewis). All patients had at least 7 months follow-up. Median follow-up was 17 months; median overall survival was not reached yet

### Operative Results

The operative results were shown in Table 2. The thoracic part of the operation was performed robotically in all patients, whereas the first 8 cases of the abdominal phase were performed with a conventional laparoscopy. Hereafter, 92 patients underwent a robotic abdominal part of the operation. Only 2 thoracic procedures were converted to an open operation: 1 due to technical problems with the creation of the gastric conduit and 1 due to massive intrathoracic adhesions in the right hemithorax. Another 2 cases were complicated intraoperatively without the need of a thoracotomy: 1 intrathoracic arterial bleeding was preserved, and a right bronchial perforation from the cuff of the double lumen tube was treated with patching of a pericardium flap.

**Table 2:** Operative details (*n* = 100)

	n = 100
Thoracic approach: RAMIE 4 (n (%))	100 (100)
Abdominal approach	
Conventional laparoscopy	8 (8)
RAMIE 4	92 (92)
Circularly stapled anastomosis	
25 mm	29 (29)
28 mm	71 (71)
Operating time (min)(mean – SD)	
Total operating time	415 ± 80
Thoracic phase operating time	213 ± 57
Abdominal phase operating time	113 ± 31
Blood loss (ml)(±SD)	
Total blood loss	311 ± 196
Conversion thoracic phase	2 (2)
Conversion abdominal phase	0 (0)
Intraoperative complications	4 (4)

Mean total operative time was 415 min (± 80 min). The mean thoracic part duration was 213 min (± 57 min); mean operating time for the abdominal phase was 113 min (± 31 min).

### Postoperative Results and Short-Term Outcome

Postoperative data are shown in Table 3. In total, 70 out of 100 patients recovered without a complication, whereas in 30 patients, the postoperative course was not uneventful. The most commonly observed complications were pulmonary complications (17%). Anastomotic leakage of the esophagogastrostomy was found in 8 patients. All patients who had anastomotic insufficiency were treated primarily with an endoscopically placed Endo-Sponge® (B. Braun, Melsungen, Germany) or a stent placement (type II anastomotic insufficiency) without a reoperation. A clinical diagnosis of paralysis of the recurrent laryngeal nerve was found in 3 patients (3%) and were unilateral and temporary. In 4 patients, a chylothorax was identified postoperatively of whom 1 patient was reoperated with thoracoscopically clipping of the thoracic duct and the other 3 were treated conservatively with dietary modifications. Thoracic wound infections were found in 2 patients. Readmittance to the ICU occurred in 7 patients due to pulmonary complication or treatment of an anastomotic leakage. One patient died within 30 days due to meningeosis carcinomatosa. (30-day mortality 1%) 90 day mortality was 3%. Median hospital stay was 11 days (range 7–92 days), and median intensive care unit (ICU) stay was 1 day (range 0–84 days).

**Table 3:** Postoperative data (n = 100)

	n = 100
Uncomplicated procedures (n (%))	70 (70)
Complicated procedures (n (%))	30 (30)
Pulmonary complications (n (%))	17 (17)
Pneumonia (n (%))	12 (12)
Pneumothorax (n (%))	2 (2)
Pleural effusion (n (%))	5 (5)
ARDS (n(%))	1 (1)
Cardiac complications (n (%))	7 (7)
Anastomotic leakage type II (n (%))	8 (8)
Chylothorax (n (%))	4 (4)
Recurrent laryngeal nerve paralysis (n (%))	3 (3)
Wound infection (n (%))	2 (2)
30-day mortality	1 (1)
90-day mortality	3 (3)
Intensive care unit (ICU) stay (days) (median – range)	1 (0–84)
Readmission ICU (n (%))	7 (7)
Hospital stay (days) (median – range)	11 (7–92)
Readmission in 30 days after discharge (n (%))	5 (5)

### Histopathological Findings and Short-Term Oncologic Results

Table 4 provided an overview of the histopathological results. The majority of the tumors were adenocarcinomas (72%). In 11 patients, no viable tumor cells were identified in the resection specimen. A median of 29 lymph nodes (range 8–65) were harvested with a median of 1 positive lymph node (range 0–33). Tumor-affected lymph nodes were found in 49 patients. Ninety-two patients had a radical (R0) tumor resection. In 8 patients, a R1 resection was performed of whom 6 had a potential positive circumferential resection margin, 1 a positive proximal resection margin, and 1 a positive distal resection margin. All patients had at least 7 months of follow-up with a median follow-up of 17 months. Median overall survival was not reached yet.

**Table 4:** Histopathological data

	n = 100
Histological type (n (%))	
Adenocarcinoma	72 (72)
Squamous cell carcinoma	15 (15)
No viable tumor cells	11 (11)
Melanoma	1 (1)
Neuroendocrine	1 (1)
Radicality (n (%))	
R0	92 (92)
Lymph nodes (number) (median – range)	29 (8–65)
Positive lymph nodes (number) (median – range)	1 (0–33)
Pathological stage (TNM 8) (n (%))	
pT0N0	11 (11)
pT0N1	1 (1)
pT1aN0	4 (4)
pT1aN2	1 (1)
pT1bN0	7 (7)
pT1bN1	1 (1)
pT2N0	9 (9)
pT2N1	4 (4)
pT2N2	1 (1)
pT2N3	1 (1)
pT3N0	18 (18)
pT3N1	9 (9)
pT3N2	14 (14)
pT3N3	14 (14)
pT4aN2	2 (2)
pT4aN3	1 (1)
pT3N1 neuroendocrine carcinoma	1 (1)
pT4bN3 melanoma	1 (1)

## Discussion

In this article, we presented the results from 100 consecutive patients who underwent RAMIE4 with intrathoracic anastomosis for esophageal cancer or cancer at the gastro-esophageal junction in Europe (the European experience). RAMIE4 was technically feasible and safe. Postoperative complications and short-term oncologic results were comparable to the highest international standards nowadays.^21^

Until now, there is 1 randomized controlled trial (ROBOT trial), which compared RAMIE to OTE.^11^ In this randomized controlled trial, all patients received a cervical end-to-side hand-sewn anastomosis (McKeown). An anastomotic insufficiency rate of 24% was observed in the RAMIE arm of this trial, which was comparable to the open arm using a cervical esophagogastric anastomosis to restore continuity.^12^ The anastomotic insufficiency rate was 8% in our study after esophagectomy with intrathoracic anastomosis (Ivor Lewis). All patients had a type II anastomotic insufficiency and were treated endoscopically with the placement of an Endo-Sponge.^15^ The percentage of anastomotic was comparable to other studies describing a robot-assisted Ivor Lewis procedure.^22–24^ These data might suggest that an (circularly stapled) Ivor Lewis anastomosis might reduce anastomotic insufficiency in patients with esophageal cancer compared to a McKeown procedure, which is in concordance with recent literature.^25^ A randomized controlled trial is currently being performed to answer the question whether a McKeown or Ivor Lewis procedure should be preferred in patients undergoing MIE.^26^

In our study, 70% of patients had an uneventful postoperative course. Pulmonary complications were observed in 17% of patients, of whom 12% had pneumonia. Chyle leaks were observed in 4% of patients, and only 1 patient had a reoperation. In 3% of patients, a recurrent laryngeal nerve injury (all temporary) was observed. These percentages were comparable to the ESODATA results which were published by the Esophageal Complications Consensus Group (ECCG), which serves as a comparison for surgical quality control.^21^ Furthermore, postoperative results were also comparable to 2 studies from the United States which compared RAMIE (Ivor Lewis) to open esophagectomy.^27,28^ In both studies, RAMIE (Ivor Lewis) demonstrated substantial benefits in postoperative complications compared to open esophagectomy. The incidence of postoperative complications observed within our series was comparable to the results obtained in aforementioned studies describing RAMIE (Ivor Lewis).^23–25,27,28^ Our results show that the results of RAMIE4 with an intrathoracic anastomosis as performed in Europe nowadays were comparable to the highest international standards worldwide.^21,27,28^ Therefore, RAMIE4 is a valuable therapeutic option for patients with resectable esophageal cancer.

Short-term oncological results were reported for patients who underwent RAMIE (Ivor Lewis) within our hospital. A radical (R0) resection was achieved in 92% of patients with a median number of 29 lymph nodes. All patients had at least a follow-up of 7 months with a median follow-up of 17 months. Until now, median overall survival was not reached yet. Short-term oncologic results and short-term survival data were comparable to studies describing (minimally in) esophagectomy.^5,8,10,22^

With proven superiority of MIE over OTE, the question remains, whether the technical advantages of RAMIE contribute to better results compared to conventional MIE. In Asian studies, where RAMIE was compared to MIE, a higher mean lymph node yield along the recurrent laryngeal nerve was observed in favor of RAMIE.^29–32^ Furthermore, RAMIE showed a reduced rate of recurrent laryngeal nerve injury compared to MIE.^33^ An improved lymph node dissection might be the result of the technical advantages or RAMIE compared to MIE.^33^ These data were in concordance with a Western study which showed improved lymph node dissection for RAMIE compared to MIE.^34^ However, randomized controlled trials might answer the question whether RAMIE is superior to MIE.

Currently, there are 2 Asian multicenter randomized controlled trials comparing RAMIE to MIE for esophageal squamous cell carcinoma patients: the REVATE trial (ClinicalTrials.gov Identifier: NCT03713749) and the RAMIE trial (ClinicalTrials.gov Identifier: NCT03094351). Results for these trials might answer the question whether RAMIE is superior to MIE in the Asian population. Within our institute, a multicenter randomized controlled trial comparing RAMIE to MIE for patients with esophageal cancer in the Western world is about to start.

Limitations of this study are the single-center design and the absence of a control group. Furthermore, quality of life and functional recovery were not recorded.

In conclusion, RAMIE with intrathoracic anastomosis for esophageal or GEJ cancer was technically feasible and safe. Postoperative complications and short-term oncologic results were comparable to the highest international standards nowadays. The superiority of RAMIE compared to MIE is currently investigated in multiple randomized controlled trials. Results of these trials will define the role for RAMIE for patients with esophageal cancer in the future.
